# Substance P participates in immune-mediated hepatic injury induced by concanavalin A in mice and stimulates cytokine synthesis in Kupffer cells

**DOI:** 10.3892/etm.2013.1152

**Published:** 2013-06-10

**Authors:** YAN YANG, MING YAN, HAITAO ZHANG, XUPING WANG

**Affiliations:** 1 Health Examination Center, Qilu Hospital of Shandong University, Jinan, Shandong 250012;; 2 Department of Gastroenterology, Qilu Hospital of Shandong University, Jinan, Shandong 250012;; 3 Interventional Center, Jinan Infectious Disease Hospital, Jinan, Shandong 250021;; 4 Key Laboratory of Cardiovascular Remodeling and Function Research, Chinese Ministry of Education and Chinese Ministry of Public Health, Qilu Hospital of Shandong University, Jinan, Shandong 250012, P.R. China

**Keywords:** concanavalin A, liver injury, substance P, neurokinin 1 receptor antagonist, neurogenic inflammation

## Abstract

Studies have indicated that the immune system plays a pivotal role in hepatitis. Substance P (SP) has been shown to modulate the immune response. In order to investigate the role of SP in liver injury and to determine whether it leads to pro-inflammatory signaling, we established a mouse model of hepatic injury induced by concanavalin A (ConA). We also exposed mouse Kupffer cells (KCs) to SP *in vitro*. Cytokine and SP levels in liver homogenates were detected using enzyme-linked immunosorbent assay (ELISA) and the protective effects of L-703,606 were evaluated through serological and histological assessments. Neurokinin-1 receptor (NK-1R) expression was evaluated by immunofluorescence and quantitative polymerase chain reaction (PCR). The levels of SP, alanine aminotransferase (ALT) and aspartate aminotransferase (AST) were significantly increased in the ConA-treated mice and the levels of ALT and AST were markedly reduced by L-703,606-pretreatment. Liver injury was significantly reduced by treatment with L-703,606. The mouse KCs expressed NK-1R and SP increased NK-1R mRNA expression. Furthermore, NK-1R blockade eliminated the effect of SP on NK-1R mRNA expression. The cytokine levels exhibited a substantial increase in the SP-pretreated KCs compared with the KCs that were cultured in control medium. The inter-leukin (IL)-6 and tumor necrosis factor (TNF)-α levels in the L-703,606-pretreated KCs were significantly lower compared with those in the SP-pretreated KCs. Our study suggests that neurogenic inflammation induced by SP plays an important role in hepatitis. Mouse KCs express NK-1R and SP increases NK-1R mRNA expression. SP enhances IL-6 and TNF-α secretion and an NK-1R antagonist inhibits this secretion.

## Introduction

Inflammatory disease of the liver, i.e., hepatitis, represents a major problem in clinical medicine. Although hepatitis comprises a heterogeneous group of diseases with different etiologies and complex pathogeneses, previous studies have indicated that the immune system plays a pivotal role in the majority of these processes (1).

Neurogenic inflammation encompasses a series of inflammatory responses triggered by the activation of primary sensory neurons and the subsequent release of inflammatory neuropeptides (2). The nervous and immune systems have been shown to interact to modulate the immune response via the secretion of neuropeptides. The neuropeptide substance P (SP) is an 11 amino acid peptide encoded by the preprotachykinin-A (PPT-A) gene, which is distributed throughout the nervous systems of humans and animals (3). SP is a member of the tachykinin family of neuropeptides, which interact with three natural tachykinin (neurokinin) receptors: NK-1R, NK-2R and NK-3R (4); however, its effects are mainly mediated through NK-1R, a G protein-coupled receptor (GPCR) that is expressed in a number of tissues, including the nervous system, gastrointestinal tract and cells of the immune system. A previous study demonstrated that SP is a potent pro-inflammatory mediator that plays an important role in inflammation and viral infections (5).

SP has been shown to exert a vast range of pro-inflammatory effects *in vitro* and *in vivo*, affecting a number of immune and inflammatory disorders of the respiratory, gastrointestinal and musculoskeletal systems. Although SP is a peptide of neuronal origin, it is also located in non-neural cells, including endothelial cells, macrophages, granulocytes, lymphocytes and dendritic cells. SP stimulates immune cells to produce inflammatory cytokines, including interleukin (IL)-1, IL-6, tumor necrosis factor (TNF), interferon (IFN)-γ and the macrophage inflammatory protein 1β. SP induces chemotaxis and degranulation of neutrophils and also stimulates respiratory burst (6).

Therefore, it is clear that extensive neuro-immune inter-system crosstalk is necessary between SP and the inflammatory response to injury. A previous study demonstrated that primary afferent sensory neurons are necessary for disease activity in T cell-mediated immune hepatitis in mice (7). Bang *et al* first demonstrated the presence of NK-1R in mouse liver, in which it appeared predominantly in nonparenchymal mononuclear cells; however, it was also detected in hepatocytes. The authors reported that NK-1R was mainly expressed in Kupffer cells (KCs), which corresponds to the cell population that is primarily activated by lipopolysaccharide (LPS) in the liver (8). However, in their study, SP was not investigated and experiments were not conducted *in vitro*. Therefore, more data are required regarding the role of SP in the liver under physiological and pathophysiological conditions. In the present study, we investigated the effect of SP in a concanavalin A (ConA)-induced model of hepatitis. We also cultured mouse KCs to examine the functional consequences of exposure to SP and to determine whether treatment with SP leads to pro-inflammatory signaling activities, including the production of cytokines.

## Materials and methods

### Drugs and materials

ConA, L-703,606, SP, collagenase IV and Triton X-100 were purchased from Sigma (St, Louis, MO, USA). Percoll, RPMI-1640 medium and fetal bovine serum (FBS) were purchased from Gibco (Carlsbad, CA, USA). The NK-1R antibody NB300-101 and rabbit anti-NK-1R secondary antibody were purchased from Novus Biologicals (Littleton, CO, USA). IL-6 and TNF-α enzyme-linked immunosorbent assay (ELISA) kits were purchased from R&D Systems (Minneapolis, MN, USA). The other commercial chemicals used in the experiments were of analytical grade.

### Animal experiments and drug treatment

Male Swiss albino mice (25–30 g) were purchased from the Experimental Animal Center of Shandong University School of Medicine, Shandong, China. The animals were housed six per cage under standardized conditions (25±3°C, 12 h light/dark cycle and 50±10% humidity) with free access to pelleted food and tap water. All experiments were conducted with approval from the Institutional Experimental Animal Care and Use Committee of Shandong University.

After acclimation for 6–7 days, the animals were randomly divided into three groups, each containing 12 mice, as follows: the control group, the ConA model group and the NK-1R antagonist group (L-703,606-pretreated group). Liver injury was induced as previously described (9). ConA was dissolved in pyrogen-free phosphate-buffered saline (PBS) and intravenously injected (25 mg/kg) into the mice of the ConA model group via the tail vein. The mice in the control group were injected with saline. The mice in the NK-1R antagonist group were treated with the NK-1R antagonist L-703,606 at dose of 10 mg/kg prior to the ConA challenge. L-703,606 was dissolved in 0.9% saline and injected via the tail vein through a 27 gauge needle. Within each experiment, all drugs and doses were administered in a counterbalanced manner. Six hours later, all mice were anesthetized to enable blood to be obtained from the eye sockets and were then sacrificed prior to dissection of the liver.

### Measurement of SP levels

The liver samples were thawed, weighed and homogenized at a ratio of 1:9 (w/v) in a 0.9% saline solution. The homogenate was then centrifuged at 1,600 × g for 10 min at 4°C. The levels of SP in the supernatant were measured using a mouse SP ELISA kit according to the manufacturer’s instructions. A 96-well microplate was loaded with 25 *μ*l primary antibody specific for rat SP. Aliquots of 50 *μ*l each sample and 50 *μ*l standard SP dilutions (as a control) were mixed in the assigned wells in duplicate, followed by the addition of biotinylated SP to each well, with the exception of the blank control. The plate was incubated for 2 h at room temperature and then washed six times with the wash buffer provided in the kit. Subsequently, 100 *μ*l biotinylated anti-SP antibody solution was added and the plate was incubated for 1 h and then washed four times. Then, 100 *μ*l streptavidinhorseradish peroxidase conjugate solution was added to each well, with the exception of the chromogen blank, and the plates were incubated for 1 h and washed again. After washing, 100 *μ*l substrate solution provided in the kit was added to each well and the plates were incubated for 1 h at room temperature. The reaction was stopped with 2 mol/l HCl and the optical density values were read at 492 nm. The concentration of SP was expressed in pg/ml.

### Measuring serum liver enzymes

Blood was collected in ethylenediaminetetraacetic acid (EDTA) tubes. Following centrifugation of whole blood (1,600 × g for 10 min at room temperature), the serum was stored at −70°C until analysis. The activities of alanine aminotransferase (ALT, a specific marker for hepatic parenchymal injury) and aspartate aminotransferase (AST, a nonspecific marker for hepatic injury) in the serum were determined in units per liter using standard auto-analyzer methods on an Hitachi Automatic Analyzer (Hitachi Inc., Tokyo, Japan).

### Histological examination

The livers were removed, fixed with 4% phosphate-buffered paraformaldehyde and embedded in paraffin. Tissue sections (4 *μ*m) were prepared and stained with hematoxylin/eosin and the sections were then examined under a light microscope. In each section, three randomly selected areas were screened for edema, granulocytes and hepatocyte apoptosis and necrosis. Histological examination was performed without knowledge of the treatment administered. The sections were examined by two independent investigators in a blind manner.

### Isolation and culture of KCs

KCs from mice were isolated by collagenase digestion and differential centrifugation using Percoll density gradients as described previously (10). Livers were perfused *in vitro* through the vena cava with 80 ml Ca^2+^/Mg^2+^-free Hank’s balanced salt solution (HBSS) at 37°C and transferred to a 100-mm culture dish. Perfusion was continued with complete HBSS containing 0.05% collagenase IV and 3 mmol/l Ca^2+^ at 37°C. The liver tissue was finely diced into 2-mm^3^-sized pieces and the suspension was incubated under constant agitation at 37°C for 30 min. The liver homogenate was filtered through a gauze mesh and the cell suspension was centrifuged at 50 × g for 3 min at 4°C to remove the hepatocytes. The non-parenchymal cell-enriched supernatant was centrifuged at 400 × g for 6 min. The cell pellet was resuspended in 30% Percoll with a density of 1.040 g/ml and this suspension was carefully layered onto 60% Percoll with a density of 1.075 g/ml. The double-layer discontinuous gradient formed was overlaid with 3 ml HBSS and centrifuged at 400 × g for 15 min at 4°C. The opaque interface was collected, resuspended in HBSS and centrifuged at 400 × g for 5 min at 4°C. The cells were seeded onto tissue culture plates at a density of 2×10^6^ cells/ml and cultured in RPMI-1640 medium containing 10% heat-inactivated FBS, 100 U/ml penicillin/streptomycin and 10 mmol/l HEPES at 37°C with 5% CO_2_. All adherent cells phagocytosed latex beads and stained positive for catalase, confirming that they were KCs. The cells were cultured for 24 h prior to the experiment.

### NK-1R immunofluorescence

Cells (5×10^4^) were placed on poly-L-lysine-coated slides in 200 *μ*l media, fixed with 4% paraformaldehyde, washed three times in 0.1 M PBS, blocked with serum-free protein and permeabilized using 0.4% Triton X-100. The fixed cells were incubated overnight at 4°C with the NK-1R antibody NB300-101 (1:50), then subjected to PBS washes and incubation with rabbit anti-NK-1R secondary antibody (1:500) for 1 h at room temperature. Then, the sections were washed in PBS and mounted with 4′,6-diamidino-2-phenylindole (DAPI)-containing mounting medium (Vector Laboratories, Burlingame, CA, USA). Images were acquired by laser scanning confocal microscopy (LSM710; Carl Zeiss, Oberkochen, Germany) and analyzed using Image Pro Plus 6.0 (Media Cybernetics Rockville, MD, USA).

### Quantitative polymerase chain reaction (PCR)

Total RNA was extracted from KCs using Tri-Reagent (Molecular Research Center Inc., Cincinnati, OH, USA) and the RNA was quantitated and reverse-transcribed using the AffinityScript qPCR cDNA Synthesis kit (Stratagene, Cedar Creek, TX, USA) according to the manufacturer’s instructions. Total NK-1R mRNA was quantified as described previously (11) using a primer set specific for a 109-bp fragment of the NK-1R transcripts (sense: 5′-GCATACACCGTAGTGGGAATC-3′; antisense: 5′-CATCATTTTGACCACCTTGC-3′). Glyceraldehyde 3-phosphate dehydrogenase (GAPDH) was amplified as a control (sense: 5′-GGTGGTCTCCTCTGACTTCAACA-3′; antisense: 5′-GTTGCTGTAGCCAAATTCGTTGT-3′). Reverse transcription (RT)-PCR was conducted with cycling conditions consisting of 15 min Taq activation at 95°C followed by denaturing, annealing and extension phases for 15 sec at 94°C, 30 sec at 54°C and 30 sec at 72°C, respectively, for 40 cycles. The results for NK-1R are expressed as the ratio to GAPDH.

### Measurement of cytokine release

Micromolar concentrations of SP are typically used during *in vitro* studies to identify the biological effects on immune cells, particularly monocytes (12). KCs were seeded into 24-well plates at a density of 1×10^6^ cells/well and incubated with fresh Dulbecco’s modified Eagle’s medium (DMEM) containing 1 *μ*g/ml LPS at 37°C with 5% CO_2_. The KCs were cultured with and without SP or with a combination of SP and L-703,606 with fresh Dulbecco’s modified Eagle’s medium (DMEM) containing 1 *μ*g/ml LPS at 37°C with 5% CO_2_ and challenged with SP (10^−6^ M) for 24 h. A dose of 1 *μ*M (10^−6^ M) SP and a stimulation time of 24 h were selected to ensure maximal cytokine release, as observed in a previous study (13). At this concentration, SP is reported to stimulate significant chemokine production by pancreatic acinar cells and prime neutrophils triggered by different stimuli to evoke various cellular responses, including intracellular calcium changes, oxidative responses and the formation of hydrogen peroxide and nitric oxide (14,15). In some experiments, the cells were pretreated with L-703,606 (1 *μ*M) for 15 min before SP stimulation, as previously described (16). The medium from the cultured KCs was collected and centrifuged at 1,000 × g for 5 min and the supernatant was stored at −70°C until the assays. The IL-6 and TNF-α levels in the supernatant were determined using mouse-specific ELISA kits. All samples, including the standard and control solutions, were assayed in duplicate. The measurements were performed according to the manufacturer’s instructions. No cross-reactivity was observed with any other known cytokine. The results are expressed in picograms per milliliter.

### Statistical analysis

All data are presented as mean ± standard error of the mean (SEM). Differences among the groups were assessed using unpaired Student’s t-tests and one-way analysis of variance. P<0.05 was considered to indicate a statistically significant difference. The calculations were performed with the SPSS, version 11.0, statistical software package (SPSS, Inc., Chicago, IL, USA).

## Results

### Detection of SP and liver function

As shown in Table I, the levels of SP were significantly increased in the ConA-treated group compared with the control group. Serum biochemical marker determinations revealed that serum ALT and AST levels were significantly increased in the ConA-treated group compared with those in the control group. However, the levels of serum ALT and AST were significantly decreased in the L-703,606-pretreated group compared with those in the ConA-treated group.

### Histopathological changes

**Figure 1.**
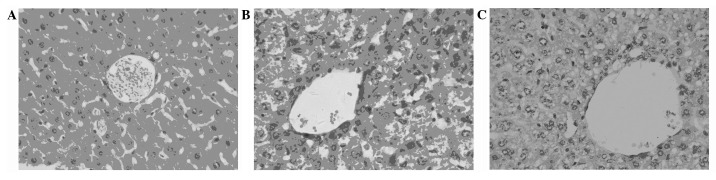
Histopathological changes. (A) No pathological changes were observed in the liver tissues of the control group (H&E staining; magnification, ×200). (B) Extensive hepatic cellular necrosis and massive cellular infiltration were observed (H&E staining; magnification, ×200). (C) Decreased hepatic necrosis was observed compared with the Con-A model group (H&E staining; magnification, ×200). H&E, hematoxylin and eosin.

A model of hepatic immune injury in mice was successfully established in this study. Our histological observations revealed that edema, hepatocellular apoptosis and granulocyte infiltration were present in the livers of the ConA model group. Compared with those of the control group (Fig. 1A), the liver tissues from the mice in the ConA-treated group exhibited significant cytoplasmic vacuolization, sinusoidal congestion, extensive hepatic cellular necrosis and massive cellular infiltration (Fig. 1B). However, the parenchymal appearance was essentially normal in the L-703,606 -pretreated group. Mild cellular infiltration, necrosis and a comparatively preserved lobular architecture were observed in the livers of mice treated with L-703,606 (Fig. 1C). The NK-1R antagonist L-703,606 was shown to significantly alleviate ConA-induced liver injury. These results suggest a therapeutic significance of L-703,606 for protection from ConA-induced liver injury.

### Mouse KCs express NK-1R and SP increases NK-1R mRNA expression in KCs in vitro

**Figure 2.**
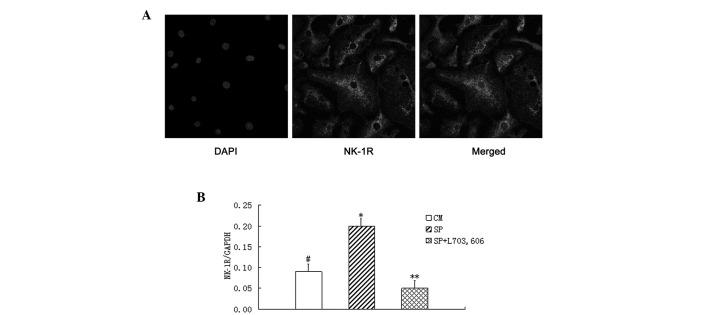
(A) Nuclei were counterstained with DAPI and the cells were imaged using fluorescence microscopy. Immunoreactivity was concentrated in the cell cytoplasm. Merged fluorescent images are shown in the third panel. The data are representative of three independent experiments. The data for the negative controls are not shown. (B) Effects of SP and L-703,606 on neurokinin-1 receptor (NK-1R) mRNA expression in KCs *in vitro*. The group data for NK-1R mRNA expression are shown. Values are mean ± SE; ^*^P<0.01 compared with CM; ^**^P<0.01 compared with SP. DAPI, 4′,6-diamidino-2-phenylindole; SP, substance P; KC, Kupffer cell; CM, control medium.

NK-1R was analyzed in KCs from mice, based on immunohistochemical localization using fluorescein. The immunoreactivity was concentrated in the cell cytoplasm (Fig. 2A), which is different from the results of other studies (8). NK-1R mRNA levels were upregulated in KCs incubated with SP for 24 h. According to quantitative PCR, the results further demonstrated that SP increased NK-1R mRNA expression 2.5-fold (Fig. 2B). NK-1R blockade eradicated the effect of SP on NK-1R mRNA expression and significantly reduced NK-1R mRNA expression to below the level observed in control culture medium.

### SP enhances IL-6 and TNF-α secretion and L-703,606 inhibits SP-induced IL-6 and TNF-α release from KCs in vitro

In order to determine whether SP-induced IL-6 and TNF-α release is mediated through the NK-1R, KCs were pre-incubated with the NK-1R antagonist L-703,606 (10 *μ*M) for 15 min and during stimulation with SP (1 *μ*M). As shown in Table II, the cytokine (IL-6 and TNF-α) levels in the super-natant resulting from the release from cultured KCs revealed a substantial increase in the SP-pretreated group compared with the control group. The IL-6/TNF-α levels of the supernatant in the L-703,606-pretreated group were significantly lower compared with those in the SP-pretreated group. In other words, the SP-induced IL-6 and TNF-α secretion from KCs was eradicated by pretreatment with L-703,606.

## Discussion

Various factors, including viral infections, autoimmune reactions and metabolic disorders are involved in liver injury. ConA-induced hepatitis, which closely mimics the pathogenesis mechanisms and pathological changes of patients, has long been regarded as an appropriate model of human immune-mediated liver disease (17). ConA is a type of lectin, which is purified from *Canavalia brasiliensis*. The mechanisms of the ConA model have interested numerous scientists; when mice are treated with ConA, lymphocytes and other mononuclear cells release lymphokines and there is a rapid inflammatory alteration of the liver tissue, including clear infiltration of neutrophils, macrophages and T cells, as well as a significant simultaneous increase in the level of aminotransferase in the peripheral blood (18). In the current study, an acute hepatitis model was successfully established (Fig. 1B).

Previous studies have demonstrated that the autonomic nervous system has a pronounced effect on immune-mediated experimental hepatitis in mice (19). It is well known that pro-inflammatory signaling mediated by SP acts through NK-1Rs (20). Therefore, since neurogenic inflammation is a result of the action of SP on NK-1Rs, we aimed to investigate whether the effects of the blockade of selective NK-1Rs using a specific NK-1R antagonist reduced the injury. L-703,606 is a selective NK-1R antagonist. Studies have verified that the effects of L-703,606 are attributable to pharmacological blockade of endogenous SP/NK-1R interactions in parallel using NK-1R −/− animals (21). In the current study, we observed that pretreatment with the NK-1R antagonist L-703,606 significantly improved liver function. This was supported by histopathological changes and our results demonstrated that disruption of the interaction between SP and its receptor had a therapeutic effect on liver inflammation. Therefore, it was concluded that SP receptor (NK-1R) antagonists are good candidates in the prevention of ConA-induced liver injury. We speculate that this protective effect may act in part via the reduction in cytokine levels. Therefore, we investigated whether SP induces KC secretion of IL-6 and TNF-α *in vitro*.

KCs are non-parenchymal cells, which account for ∼15% of the total liver cell population and constitute 80–90% of the tissue-resident macrophages in the whole body (22–23). KCs represent the major source of inflammatory cytokine production and thus the systemic release of pro-inflammatory mediators (24). There is considerable evidence indicating that the activation of KCs and their production of pro-inflammatory cytokines contribute to the pathogenesis of various liver injuries, including alcoholic liver disease (ALD), non-alcoholic fatty liver disease (NAFLD) and liver failure (25). The activation of KCs results in the release of an array of inflammatory mediators, growth factors and reactive oxygen species, including TNF-α and IL-6, which contribute to hepatocellular damage (26). Furthermore, accumulating evidence indicates that inactivation of KCs prevents liver injury (27). Therefore, the factors that control the KC response are clearly critical in the progression of liver injury. SP has been shown to affect cytokine production and release from various immune cells. For example, SP modulates the release of IL-1, IL-6 and TNF-α from human blood monocytes (12). In monocytes and macrophages, SP also stimulates the release of arachidonic acid metabolites and pro-inflammatory cytokines; it induces the respiratory burst and acts as a potent chemoattractant (28). SP has been detected in the liver, and receptors for SP have also been detected on KCs and hepatocytes. However, there is limited information concerning the correlation between SP and cytokines in KCs. Previous studies have indicated that NK-1Rs are upregulated at sites of inflammation in a number of tissues, including joints and the intestine (29–31). The current study demonstrates that mouse KCs expressed NK-1R and that treatment with SP increased NK-1R mRNA expression in KCs *in vitro*. However, at this early stage, the cytokine (IL-6 and TNF-α) levels in the supernatant resulting from the release from cultured KCs without LPS were below the minimum detection range (data not shown). Although LPS was involved in the experiment, it did not affect our study since the three groups of KCs were all incubated with it. The cytokine (IL-6 and TNF-α) levels are shown in Table II. To the best of our knowledge, we report for the first time that SP induces IL-6 and TNF-α production in KCs. In the current study, L-703,606 exhibited strong activity by reducing the production of TNF-α and IL-6 in SP+LPS-stimulated KCs, suggesting that the inhibition of pro-inflammatory cytokine (TNF-α and IL-6) release by KCs may contribute to L-703,606-mediated liver protection. Further *in vivo* and *in vitro* studies are required to determine the exact molecular pathways affected by L-703,606, which may be useful in the development of novel treatments for hepatic injury and fibrosis.

In summary, the present study and our previous findings indicate that inflammatory cytokine-mediated apoptotic liver injury is affected by neuropeptides. NK-1R agonists, including SP appear to be major players in this scenario by upregulating the pro-inflammatory cytokine response. If the data from our mouse experiments on the hepatoprotective potential of NK-1R antagonists is transferred to clinical practice, it is likely to have potential clinical implications, providing a future strategy for therapeutic intervention in the treatment of liver injuries through the suppression of KC activation and pro-inflammatory cytokines. Moreover, these findings may offer novel therapeutic targets for ameliorating liver fibrosis and cirrhosis progression. In future experiments we aim to investigate whether SP is as important in chronic liver injury as it is in acute liver injury; this may provide new avenues of investigation for the treatment of acute and chronic liver inflammation. Specifically, further studies focusing on whether SP plays a role in other biological processes of KCs, including proliferation, survival and migration, are required to improve our understanding.

## Figures and Tables

**Table I.** Levels of SP and enzymatic markers of liver function in the different groups.

Groups	ALT (U/l)	AST (U/l)	SP (pg/ml)
Control group	42.05±8.31	51.12±9.16	138.52±13.23
ConA model group	782.37±21.51^a^	1004.25±18.24^a^	387.23±29.36^a^
L-703,606-pretreated group	402.22±16.42^b^	581.45±17.51^b^	

Results are presented as mean ± standard error of the mean (SEM; n=12 mice per group).

a P<0.05 vs. the control group;

b P<0.05 vs. the ConA model group. SP, substance P; ALT, alanine aminotransferase; AST, aspartate aminotransferase; ConA, concanavalin A.

**Table II.** IL-6 and TNF-α levels in the different groups.

Group	IL-6 (pg/ml)	TNF-α (pg/ml)
Control group	71.13±9.36	31.02±7.26
SP-pretreated group	194.12±11.09^a^	92.15±8.56^a^
L-703,606-pretreated group	68.16±9.51^b^	28.38±5.04^b^

Values are presented as mean ± standard error (SE).

a P<0.05 compared with the control group;

b P<0.05 compared with the SP group. SP, substance P; IL, interleukin; TNF, tumor necrosis factor.

## Abbreviations:

Con A: concanavalin A;
SP: substance P;
NK-1R: neurokinin 1 receptor;
KC: Kupffer cell;
IL: interleukin;
TNF-α: tumor necrosis factor α
